# Novel nanoscale bacteriophage-based single-domain antibodies for the therapy of systemic infection caused by *Candida albicans*

**DOI:** 10.1038/srep32256

**Published:** 2016-08-25

**Authors:** Shuai Dong, Hongxi Shi, Donghui Cao, Yicun Wang, Xintong Zhang, Yan Li, Xiang Gao, Li Wang

**Affiliations:** 1 Institute of Cytology and Genetics, School of Life Sciences, Northeast Normal University, Changchun City, Jilin Province, 130024 P. R. China; 2 Division of Clinical Epidemiology, First Hospital of Jilin University, Changchun City, Jilin Province, 130021 P. R. China; 3 Key Laboratory of Molecular Epigenetics of MOE, Changchun City, Jilin Province, 130024 P. R. China

## Abstract

*Candida albicans (C. albicans*) is an important human commensal and opportunistic fungal pathogen. Secreted aspartyl proteinases (Saps) are a major virulence trait of *C. albicans*, and among these proteases Sap2 has the highest expression levels. It is possible that antibodies against Sap2 could provide an antifungal effect. In this study, two phages displaying anti-rSap2 single chain variable fragments (scFvs) were screened from human single fold scFv libraries, and their potential therapeutic roles were evaluated using a murine model infected by *C. albicans*. The *in vivo* efficacies were assessed by mortality rates, fungal burden and histological examination. Overall survival rates were significantly increased while the colony counts and infectious foci were significantly decreased after treatment with the scFv-phages relative to the control groups. In order to investigate the immune response provoked by scFv-phages, three kinds of cytokines (Th1, Th2 and Th17 types) were measured and a clear immune response was observed. These findings suggest that anti-rSap2 scFv-phages have potential in the therapy of systemic infection caused by *C. albicans*.

*Candida* species are the most common human fungal pathogens, and rank as the fourth most common cause of nosocomial bloodstream infections^1^. Systemic candidiasis is associated with high mortality when the fungi infect the bloodstream (called candidemia) and subsequently invade internal organs^2^. Among the *Candida* species, *Candida albicans (C. albicans*) represents the most prevalent in systemic infections, accounting for approximately 50% of cases of candidemia. It is an opportunistic fungal pathogen, which colonizes the skin and mucosal surfaces in healthy subjects, and can cause hospital-acquired bloodstream infections in immunocompromised hosts^3^,^4^, leading to mortality rates as high as 81% because of poor treatment options^5^,^6^,^7^. According to WHO statistics, Candida infections add a total of US$ 8 billion to US health-care expenditures every year^8^.

Currently, even after treatment with classical antifungal drugs, mortality caused by Candida infections remains high, in part because of adverse drug reactions, side effects, and the high cost of treatment^9^,^10^. The increasing incidence of drug resistance has hindered the development of new antifungal agents in clinical therapy^11^. Recently, some evidence suggested that antibodies could be regarded as therapeutic agents against various fungal infections including *C. albicans*^12^. Specifically targeting virulence factors, such as the secreted proteases and surface antigens specific to dimorphic forms, have been considered as attractive strategies^13^,^14^. However, the preparation of animal-derived monoclonal antibodies is laborious and expensive. Combinatorial phage-antibody libraries, in which scFv fragments are expressed as a fusion with several kinds of M13 phage coat proteins, have emerged as an excellent tool to generate antibodies compared with the traditional methods. Phage display and antibody technology has the specificity of selecting single human monoclonal antibodies against immunodominant targets^15^. It can be controlled through genetic engineering and chemical modification^16^. Mice infected with vancomycin-resistant *Enterococcus faecium* could be treated by phage, which played the role of antibacterial reagents^17^. Currently, the results of clinical trials indicated that phage had no acute toxicity^18^, and phage-displayed scFvs have been proved to be stable and can be produced rapidly, implying that they have the potential to be developed into therapeutics^19^.

The secreted aspartyl proteinases (Saps) of *C. albicans* are encoded by a family of 10 *SAP* genes^20^ and have been considered as key virulence determinants of *C. albicans.* They are clustered into three distinct groups, each of which are characterized by close sequence homology and physiological relevance^21^. Among the Sap family, Sap2 is up to 67% identical to Sap1 and Sap3, and it is the most highly expressed and the major cause of damage and virulence when infected the host^21^,^22^,^23^. Sap2 is critical for mucosal infections and probably contributes to systemic infections, it hydrolyses proteins of the immune system, and finally Sap2 penetrates the host tissue and degrades many human proteins^24^,^25^,^26^. Hube *et al.* reported that the virulence of a *C. albicans sap2* mutant strain was reduced significantly in an infection model^27^. Thus, a protective effect of a Sap antibody opens up a new way to investigate the therapy of the *C. albicans* infections^28^.

In this study, two anti-rSap2 scFvs were identified through screening of human single fold scFv libraries, and both of the novel nanoscale anti-rSap2 scFv-phages could inhibit fungal colony counts and infectious foci through mediating immune response. Moreover, the survival percentage of scFv-phage-treated Candida-infected animals was increased. Therefore, these novel therapeutic materials combine the advantages of bacteriophage and single chain variable fragments, to provide a potential candidate for the therapy of systemic infection caused by *C. albicans*.

## Results

### Screening and characterization of scFv-phages binding to rSap2

**Figure 1:**
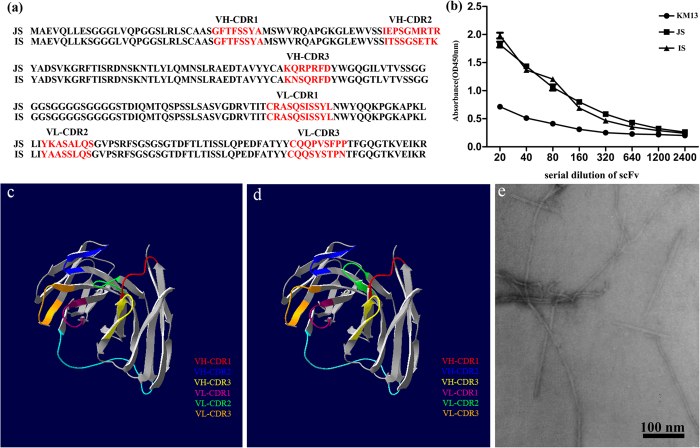
Amino acid sequences of selected scFvs and the binding ability of scFv-phage JS and IS with rSap2. (**a**) Sequences were listed starting with the initiation methionine. VH and VL were variable regions of the heavy chain and light chain. Red fonts indicated complementary determinant regions (CDR) of VH and VL. (**b**) The samples were serially diluted against rSap2 in ELISA. The absorbance at OD 450 nm was measured and the results were calculated as the mean values with SD. The three-dimensional structure of the scFv molecule and the predicted antigen-binding site of JS (**c**) and IS (**d**), were analyzed by DeepView. CDR: complementarity determining region; VH and VL: heavy and light chains, respectively. (**e**) A transmission electron microscopy (TEM) image of the stained phages.

The high binders were sought from human single fold scFv libraries I+J. They were used on the screening of hapten peptide (histamine-succinyl-GSYK, Him), a universal reporter system in a wide range of therapeutic and imaging applications^29^. In this study, the coating antigen was recombinant protein Sap2 (rSap2), which was purified and could be recognized by an anti-His-tag monoclonal antibody (Fig. S1). After 3 rounds of regular bio-panning with rSap2, phages with strong affinities and slow off-rates were obtained. 70 clones were sequenced, 43 and 27 full-length scFvs clones were found in library J and I, respectively. Interestingly, two separate clones dominated the population of selected clones (43/70, and 19/70) and were designated as JS and IS according to their screening source. The amino acid sequences of selected scFvs are listed in Fig. 1a.

A scFv represents the smallest functional domain of an antibody providing antigen-binding activities^30^. It has heavy (VH) and light (VL) chains attached to one another by a flexible Glycine-Serine linker, with three variable complementarity determining regions (CDR regions) inserted into the conserved framework regions in both VH and VL. It is noteworthy to point out that the CDR regions of JS and IS consisted of a high percentage of polar amino acids (asparagines, cysteine, glutamine, serine, threonine, tyrosine); 44.2% and 61.5%, respectively. Comparatively, the percentage of hydrophobic amino acids (alanine, isoleucine, leucine, phenylalanine, tryptophan, valine) was 25% and 21.2%, respectively (Table S1). These results suggest that the scFvs have good hydrophilic properties. The amino acid sequences of JS and IS were submitted to DeepView to predict their 3D structures and identify the CDR regions, 3 VH-CDRs and 3 VL-CDRs were observed (Fig. 1c,d). In indirect ELISA, the titers of JS and IS were determined with rSap2 at the dilution of 1:2400, whereas helper phage KM13 was relatively poorly recognized (Fig. 1b). Figure 1e shows a transmission electron microscopy (TEM) image of the stained phage.

### Binding specificity of scFv-phage antibodies

**Figure 2:**
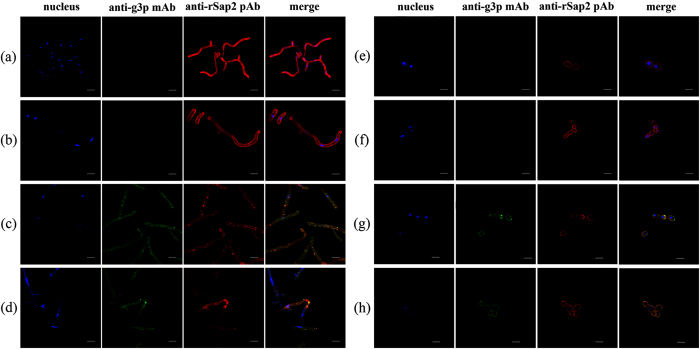
The scFv-phages and poly-antibodies were used for indirect immunoinfluscent assay of *C.* *albicans* yeast cells and hyphal cells. PBS and KM13 binding to *C. albicans* are shown in (**a,e**) and (**b,f**), there were no detectable fluorescence, which were used as controls. The positive control was anti-Sap2 pAb. It was confirmed that JS (**c,g**) and IS (**d,h**) could not only bind to yeast forms, but also were found in hyphal forms of *C. albicans*. The slides were observed under a confocal microscope using the blue, green and red light channel. Blue, DAPI. Green, FITC-conjugated goat anti-mouse IgG. Red, PE-conjugated goat anti-rabbit IgG. Scale bar, 5 μm.

The two anti-rSap2 scFv-phages were tested for their ability to recognize *C. albicans* cells. We first observed the co-localization of the scFv-phages and *C. albicans* cells. After fixation, *C. albicans* cells in two morphologic stages were incubated with PBS, KM13, JS and IS scFv-phages, respectively. Immunofluorescence analysis showed that extant signals were observed in JS and IS incubation groups compared with the PBS and KM13 incubation groups, in which no fluorescence signal was detected. Interestingly, the localization of JS and IS was not only restricted to the cell membrane surface but also observed in the cytosol of both yeast cells and hyphal cells (Fig. 2). Furthermore, the scFv-phages also had strong binding ability to the Sap2 protein as indicated by the reaction of polyclonal antibody against rSap2 (anti-rSap2 pAb).

The reactivity of scFv-phages to rSap2, *C. albicans* total cell lysates, and whole cell wall lysates was analyzed by Western blotting to determine their specificity. The results showed that anti-rSap2 scFv-phage gave a specific and robust signal for both rSap2 and native Sap2 in total cell lysates of *C. albicans*. However, another two bands were detected in total cell lysates, which were also observed when reacted with anti-rSap2 pAb. These indicated that the scFv-phages might only target Sap2, and the two lower molecular weight bands might be the autodegradation components of Sap2 as reported before^31^. Moreover, the cross reaction of anti-rSap2 scFv-phages against cell wall lysates was also excluded as no band was observed (Fig. S2).

### Effects of anti-rSap2 scFv-phages on the systemic *C. albicans* infection

In order to evaluate side-effects of phage on host, the helper phage KM13 was injected intravenously into mice, and the contents of white blood cells (WBC), lymphocytes (LY), neutrophils (NE), monocytes (MO), the red blood cells (RBC), hemoglobin (HGB) and blood platelets (PLT) in the blood were investigated. As shown in Table 1, no significant differences between the treatment and PBS groups were observed, implying that no haematological system diseases, bacterial infections and hypersplenism were caused by KM13, and medullary hematopoiesis function was normal. Thus, phage could be utilized *in vivo* in the subsequent study.

**Figure 3:**
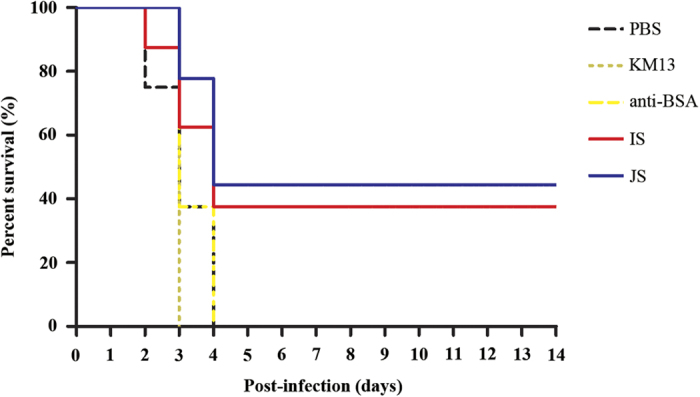
The survival percentage of mice challenged with *C. albicans* and treated with scFv-phages. The mice were challenged with 1 × 10^7^ *C. albicans* cells and treated with scFv-phages or controls at the indicated concentrations (1 × 10^7^ pfu JS and IS). The number of living mice was monitored daily for 14 days. The survival percentage of scFv-phages treated animals was higher than that of control mice, as assessed by Log-rank test (n = 8).

In order to elucidate the effects of anti-rSap2 scFv-phage on systemic *C. albicans* infections, infected mice were administered with PBS, KM13 and anti-BSA scFv-phage as negative controls, in which a higher mortality was observed compared with the JS and IS treatment groups (14-day survival, 44.4% versus 0%, JS versus PBS, respectively; *P* = 0.19) (Fig. 3).

**Figure 4:**
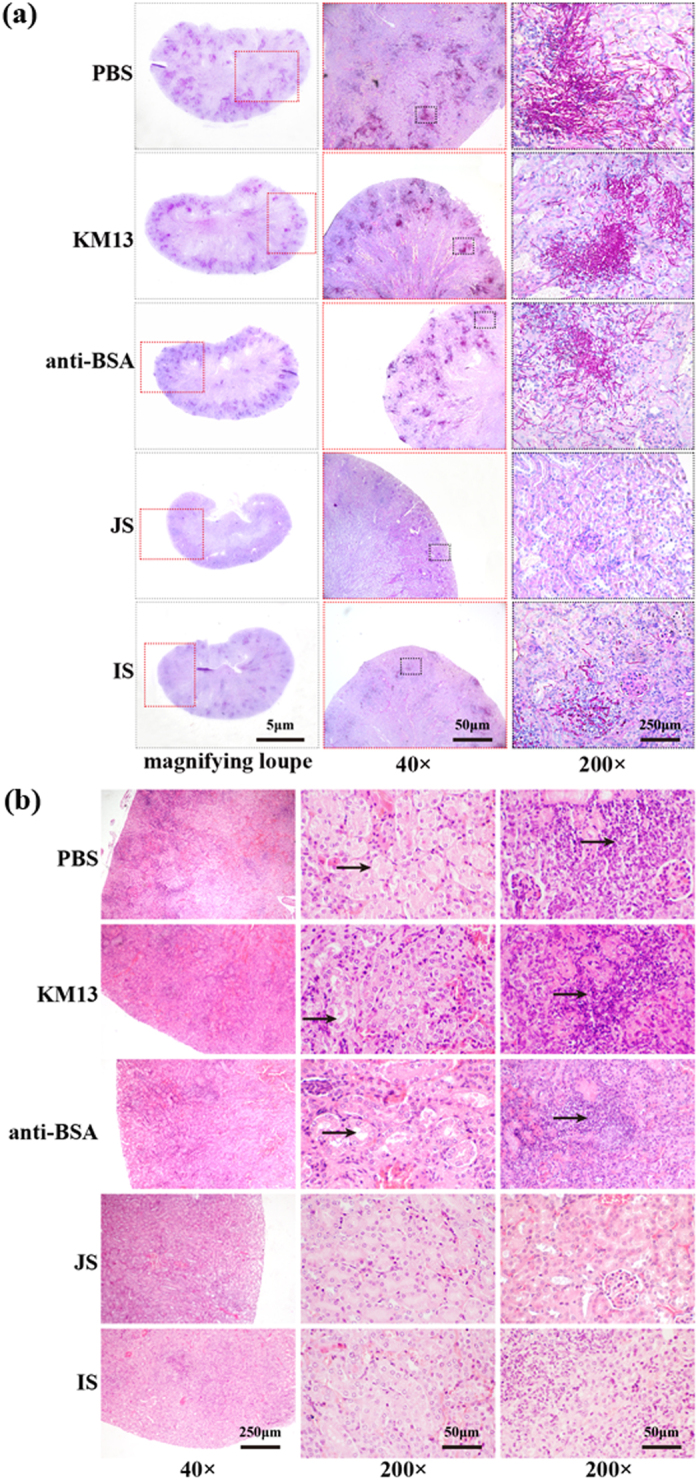
Representative periodic acid-Schiff (PAS) or hematoxylin and eosin (HE)-stained histological sections of the kidneys from mice with systemic candidiasis. Kidneys of injected mice were taken, fixed, embedded in paraffin wax, and stained with PAS (**a**) or HE (**b**). Right panel showed an enlarged image of the left rectangle. Arrows indicated a tubular cavity representing for the characteristic vacuolation and lymphocytic infiltration.

Light-microscopic examinations of PAS-stained and H&E-stained kidney-tissue sections of mice with disseminated *C. albicans* infection were carried out to investigate the size and number of infectious foci. In addition, the morphology of *C. albicans* in these tissue sections was also studied. In control groups, a large number of vacuolation and lymphocytic infiltrations were found (arrow, Fig. 4b), and multiple foci were observed in fungal cells (Fig. 4a) after 3 days of injection. Additionally, both kinds of *C. albicans* cells (yeast and branched hyphae) were observed and were distributed more in cortical regions than medullar regions. In contrast, only few vacuolation and lymphocytic infiltrations were observed in mice treated with JS and IS, and the number of infectious foci as well as *C. albicans* germ-tube formation also decreased.

**Figure 5:**
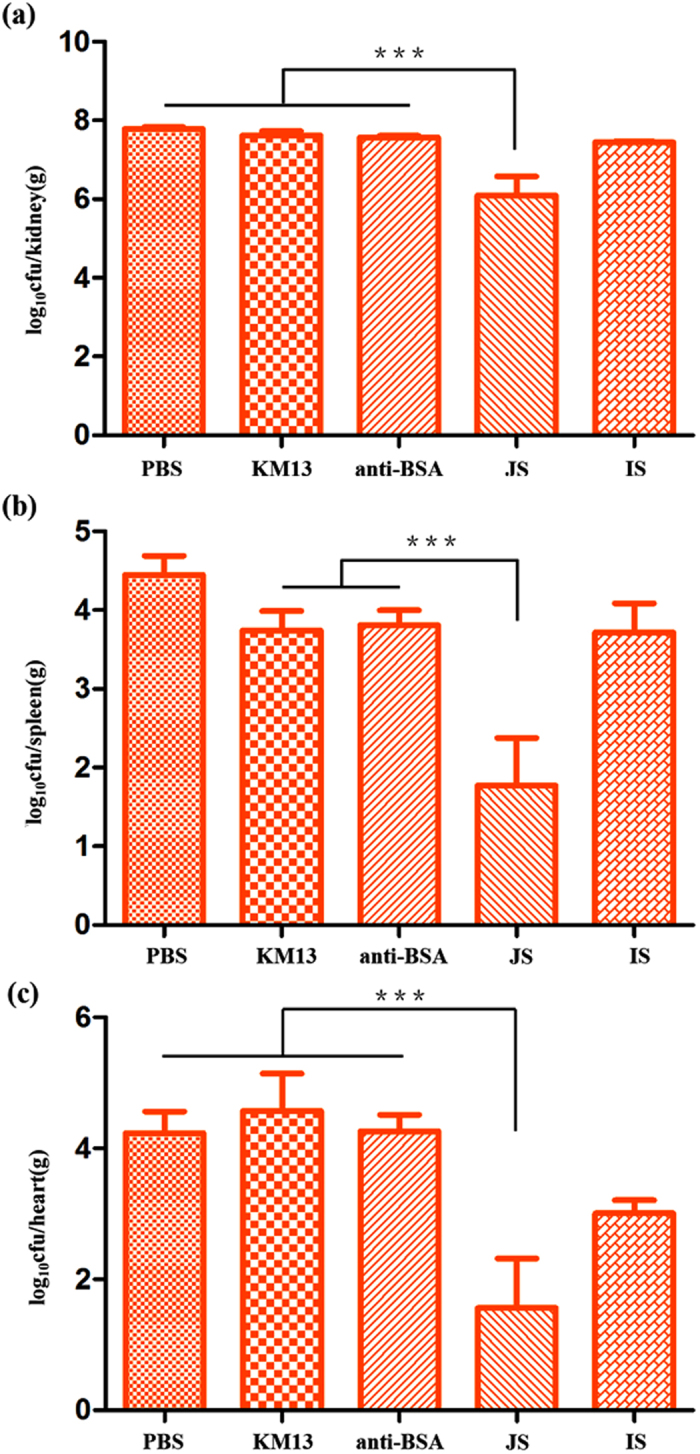
The determination of fungal clearance from organs treated with anti-rSap2 scFv-phages against infected mice. The mice were infected with 1 × 10^6^ cells of *C. albicans* by the intravenous route. 1 × 10^7^ pfu JS or IS was therefore administered intravenously 2 h after the infection, followed by a repeat treatment given at 24 h. PBS, KM13 and anti-BSA phages were served as negative controls. Mice were killed at 3 days post-infection. The fungal burden in the kidneys (**a**), spleens (**b**) and hearts (**c**) of *C. albican*s-infected mice treated with PBS, KM13, anti-BSA, JS or IS were shown. The results were expressed as mean ± SD (n = 5). Each graph was representative of three independent experiments. *P < 0.05; **P < 0.01; ***P < 0.001.

To determine the effect of JS and IS on the outcome of *C. albicans* infection, BALB/c mice infected with *C. albicans* were used to examine the fungal burden in kidneys, spleen and hearts (Fig. S3). After 3 days of treatment with JS and IS, the fungal burden in the three organs decreased compared with control groups, with the influence of JS more effective (Fig. 5a–c), especially in the heart. In conclusion, both anti-rSap2 scFv-phage played an important role against *C. albicans* infection in mice, especially for JS which had a better efficacy.

### Effects of anti-rSap2 scFv-phage on the immune system response

**Figure 6:**
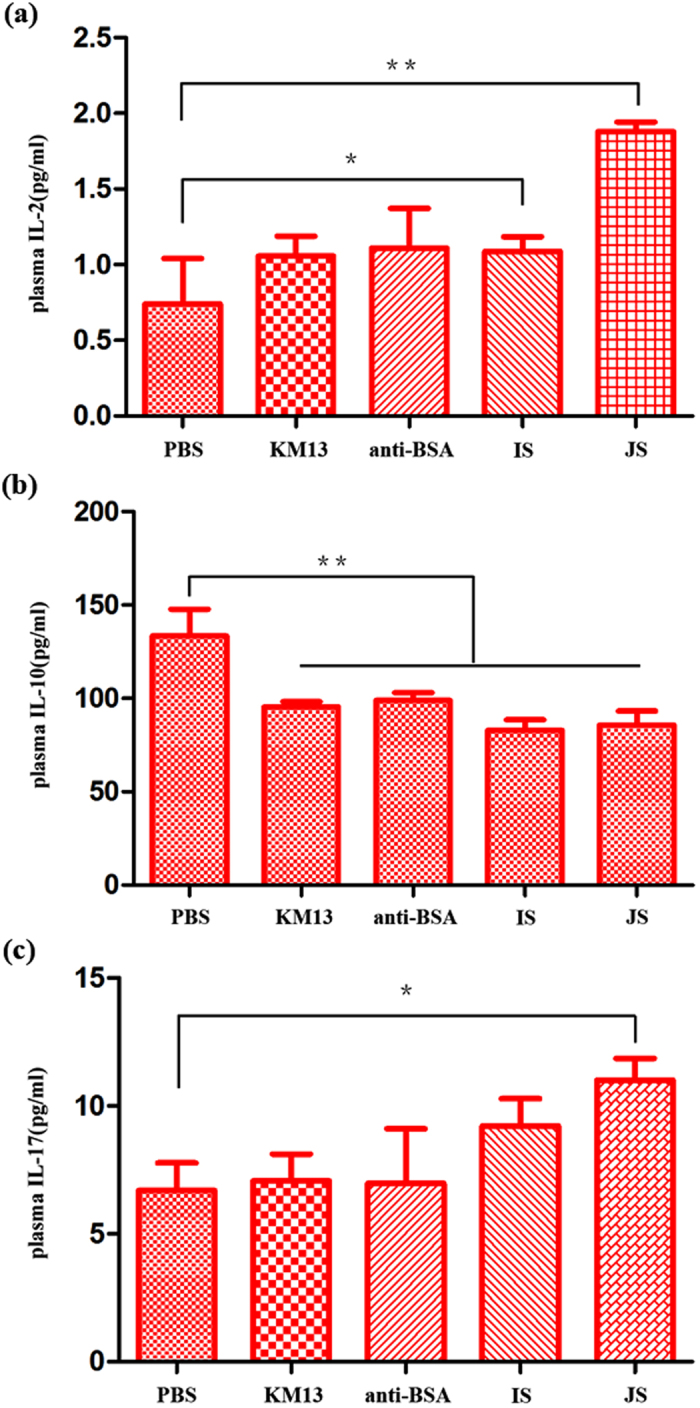
The determination of cytokine responses in *C. albicans*-infected mice treated with anti-rSap2 scFv-phages. The mice were infected with 1 × 10^6^ cells of *C. albicans* by the intravenous route. 1 × 10^7^ pfu JS or IS was therefore administered intravenously 2 h after the infection, followed by a repeat treatment given at 24 h. PBS, KM13 and anti-BSA phage were served as negative controls. The blood samples were collected at 3 days after infection. The Th1 (IL-2) (**a**), Th2 (IL-10) (**b**), Th17 (IL-17) (**c**) cytokines were measured by ELISA. The results were expressed as mean ± SD (n = 5). Each graph was representative of three independent experiments. *P < 0.05; **P < 0.01; ***P < 0.001.

For determining how the immune response was modulated by scFv-phage, the levels of several cytokines, i.e. interleukin-2 (IL-2), interleukin-4 (IL-4) and interleukin-17 (IL-17), were examined in the serum of administered mice. The results showed that the concentration of IL-2 and IL-17 was statistically increased in the groups treated with JS after infection, whereas the level of IL-4 was significantly decreased (Fig. 6a–c). In addition, IL-2 was also induced significantly after IS treatment. When compared, the change of cytokines in the IS treatment group was less significant than for JS.

## Discussion

This study presents a potential new strategy in systemic candidiasis therapy. The efficacy and the number of antifungal drugs is limited because of characteristics such as cytotoxicity and host drug resistance. It has been verified that passive transfer of anti-Sap2 immunoglobulin G (IgG) to *C. albicans*-infected mice could significantly decrease the yeast burden of kidneys^32^. Cassone *et al.* reported that anti-Sap2 antibodies selected from a human Fab antibody library could contribute to control vaginitis^33^. Recently, our studies have further shown that mice infected with *C. albicans* could get significant passive protection after inoculation of purified Sap2 polyclonal antibody^34^. Although phage display antibody library method represents an excellent tool to strengthen the treatment of invasive fungal infections, no therapeutic agents based on the human antibody library against Sap2 have been reported to control systemic candidiasis in experimental models.

To our knowledge, two of the anti-rSap2 scFv-phages screened from human single fold scFv libraries were the first proved to be recognized specifically by rSap2. Moreover, the impact of these two scFv-phages on *C. albicans* systemic infection was evaluated in an *in vivo* model, and a remarkable therapeutic efficacy was found, implying their potential in the therapy of systemic candidiasis. First, mice treated with scFv-phages exhibited a longer survival time. Second, few vacuolation and lymphocytic infiltrations were observed in kidney-tissue sections of mice treated with JS and IS. Third, the fungal burden in three organs (kidneys, spleens, hearts) of mice injected with anti-rSap2 scFv-phages were significantly decreased compared with the control groups.

Previous studies demonstrated that the important lines of defence against candidiasis were cell-mediated immunity and innate immunity^12^. Th1 helper cells are the host immunity effectors against intracellular bacteria and protozoa, and the Th1-mediated response can protect the host against pathogenic fungi, such as *C. albicans* infections^35^,^36^. IL-2, a kind of Th1-type cytokine, has been found to be able to inhibit and attack the growth of *Cryptococcus neoformans* without major histocompatibility complex (MHC) restriction^37^ through activating human lymphocytes. Th2 cells induce the humoral immune system, particularly for IL-10, suppresses Th1 cell differentiation and the function of dendritic cells. The interaction between cytokines from the Th1/Th2 balance is complicated; Th1-type cytokines are required for clearance of a fungal infection, while Th2-type cytokines usually result in susceptibility to infection or allergic responses^38^. The decreasing of IL-10 drives CD4^+^T cells differentiating into Th1 cells, which can yield more Th1 cytokines, such as IL-2. As a result, the host immunity defence shifts from Th2-type to Th1-type. Th17 helper cells are a subset of T helper cells developmentally distinct from Th1 and Th2 lineages, which produce interleukin 17 (IL-17), interleukin 6 (IL-6)*, etc*. The Th17 response is crucial for anti-*C. albicans* host response and plays an important role in clearance of various pathogens^39^,^40^. In this study, Th1 and Th17 cytokines were significantly increased in mice treated with anti-rSap2 scFv-phages, which meant the cellular immunoresponse may represent one of the therapeutic mechanisms *in vivo*. Our previous studies also showed that the hybrid phage displaying the epitope of Sap2 SLAQVKYTSASSI was a potential vaccine against *C. albicans* infection, potentially through triggering immunoprotection against fungal infection. It probably fulfilled a major role in the cell-mediated immunity response, as well as providing protection to mice against very high doses of *C. albicans* challenge^41^.

In conclusion, anti-rSap2 scFv-phage played an efficient therapeutic role in candidiasis, especially for JS. Therefore, the use of anti-rSap2 scFv-phages may provide a new platform for the treatment against candidiasis. Our methodology can also be further exploited to develop specific antibodies against other Candida protective antigens through screening human single fold scFv libraries.

## Materials and Methods

### Ethics statement

All animal experiments were performed in agreement with the recommendations in the Guide for the Care and Use of Laboratory Animals of China Association for Laboratory Animal Science. The Ethics Committee of Northeast Normal University approved all animal care and protocols (Changchun, China).

### Materials

*C. albicans* strain ATCC10231 was used in this study. The pET28a*-Sap2* recombinant plasmid was constructed and transformed into the competent cell of BL21 (DE3) to generate recombinant Sap2 fusion protein (rSap2) with a His-tag sequence, which was purified through an affinity chromatographic column of Ni-NTA (GE Healthcare Life Sciences, USA). Polyclonal antibody against rSap2 (anti-rSap2 pAb) was produced as described previously^42^ and conserved in our laboratory. Female BALB/c mice, 6–8 weeks of age, were purchased from Beijing HFK Bioscience Company and maintained in the Specific-Pathogen-Free Animal Facility of Jilin University, China.

### The screening of human single fold scFv libraries I+J

Human single fold scFv libraries (Tomlinson I + J) (in phagemid/scFv format-fused to the pIII minor coat protein of M13 bacteriophage) were purchased from MRC (Cambridge, England). The procedure of bio-panning was the same as described previously^43^ with some modifications. In brief, 4 ml of the required antigen was coated on immunotubes (Nunc Maxisorp, Scotland, UK) overnight. The concentration of rSap2 was 100 μg/ml in first round, and then gradually decreased to 50 μg/ml and 25 μg/ml in the following two rounds, respectively. On the following day, immunotubes were filled to brim with 2% MPBS (2% Marvel milk powder in PBS), and incubated at room temperature (RT) for 2 h to block. Then 10^12^ to 10^13^ phage was added into 4 ml of 2% MPBS and incubated for 60 min at RT. The unbound phage was removed by washing 10 (round 1) to 20 (rounds 2 and 3) times with PBS (pH 7.2) containing 0.1% Tween 20. The bound phage was eluted using 500 μl trypsin-PBS (1 mg/ml) for 10 min. Half of the eluted phage was used to infect a fresh exponentially growing culture of *E. coli* TG1 cells (OD_600_ = 0.4), and incubated for 30 min at 37 °C in a water bath without shaking. The rescued phage particles were used for the further screening rounds. 3 rounds of bio-panning experiments were carried out for the screening of scFv-phage clones with specific binding to rSap2.

### Preparation and characterization of the scFv-phage

The positive JS and IS phage isolated from human single fold scFv libraries were used to infect *E. coli* TG1. After inoculating a single plaque into 5 ml of fresh TG1 medium at an OD_600_ of 0.4, the cells were grown overnight shaking at 37 °C, 50 μl of which was then added to 50 ml 2 × TY for amplified culture at the same conditions for 2 h. 10 ml of this culture was added with 5 × 10^10^ helper phage, and incubated in a 37 °C water bath for 30 min statically. After centrifugation at 3,000 × g for 10 min, the pellet was resuspended in 50 ml of 2 × TY containing 100 μg/ml ampicillin, 50 μg/ml kanamycin and 0.1% glucose, and incubated at 30 °C overnight. The overnight culture was centrifuged at 3,300 × g for 15 min and 40 ml of supernatant was added to a 10 ml PEG/NaCl solution (20% Polyethylene glycol 6000, 2.5 M NaCl). The mixture was kept in ice for 1 h and spun at 3,300 × g for 30 min, then the pellet was resuspended in 2 ml PBS and spun at 11,600 × g for 10 min to remove the remaining bacterial debris.

Phage JS and IS were negatively stained with uranyl acetate. Briefly, a drop of liquid sample was transferred onto a dry copper grid which was previously coated with carbon, stained with 2% uranium acetate, and examined by transmission electron microscopy (TEM).

### Confocal laser-scanning microscopy

The yeast cells were incubated in yeast extract peptone dextrose (YPD) medium. In order to obtain hyphal cells, 1 × 10^6^ cells in RPMI 1640 medium were incubated at 37 °C for 90 min. Cells were washed 3 times with PBS (pH 7.4), and fixed with 4% paraformaldehyde for 30 min. The mixture was incubated at RT with JS or IS (2.5 × 10^9^ pfu) for 1 h, permeabilized in 0.5% Triton X-100 for 30 min and blocked with 3% BSA for 1 h. Then anti-rSap2 pAb and anti-phage g3p (pIII) antibody (Mo Bi Tec, Germany) were used, respectively. Firstly, anti-rSap2 pAb (1:1000) was incubated for 1 h, then, the cells were washed 3 times and incubated with anti-phage g3p (pIII) antibody diluted 1:1000 in PBS (pH 7.4) for 1 h at 4 °C. Next, cells were washed and stained with a secondary FITC-conjugated antibody and phycoerthrin (PE) conjugated antibody (ZSJQ-Bio, Beijing, China) for 1 h, followed by DAPI (1:200) (ZSJQ-Bio, Beijing, China) staining for 10 min. Fluorescence was detected by Olympus BX50 FluoView confocal microscope, with a 63 × NA 1.4 oil-immersion objective.

### Preparation of *C. albicans* cell walls and Western blotting analysis

The *C. albicans* cell pellet was harvested by centrifugation, and cell walls were prepared as described elsewhere^44^. Briefly, the cell pellet was washed 3 times in PBS (pH 7.4) and then centrifuged, then ground to a fine powder in liquid nitrogen at the presence of a protease inhibitor cocktail. Isolated cell walls were washed 3 times with MilliQ water and then boiled for 10 min in SDS extraction buffer [150 mM NaCl, 2% (w/v) SDS, 100 mM Na-EDTA, 100 mM beta-mercaptoethanol, 50 mM Tris/HCl, pH 7.8]. Subsequently, they were washed again with MilliQ water and stored at −20 °C until required.

Equal amounts of proteins of rSap2, cell walls and total cell lysates were separated by SDS-PAGE (10%), and transferred to a PVDF membrane (Millipore, Germany) using a blotting system (Bio-Rad, USA) for 1 h at 90 V. The membrane was incubated with the scFv-phages (1 × 10^10^ pfu), anti-rSap2 pAb and KM13 phages respectively, and subsequently blotted with different secondary antibodies, i.e., anti-phage g3p antibody for phages and goat anti-rabbit IgG for anti-rSap2 pAb. Detection was achieved with the chemiluminescence method according to the manufacturer’s instructions (Millipore, Germany), and visualized by using the ECL detection system (Tanon 5500, Shanghai, China).

### Construction of infection models of systemic candidiasis

To verify the toxicity of the phage, female BALB/c mice were infected intravenously with 1 × 10^7^ pfu helper phage KM13, and repeat infection was performed on days 7, 14, and 21. Blood samples were collected at the end of day 21 and kept in vacuum tubes containing K3 EDTA. The contents of WBC, LY, NE, MO, RBC, HGB and PLT in the blood were determined using an automatic blood analyser (Blood Analysis System XFA6100, Jiangsu, China).

*C. albicans* strain ATCC 10231 was grown in YPD broth at 37 °C overnight. Then cells were washed and resuspended in PBS. For survival studies, BALB/c mice of SPF level 6–8 weeks old and 18–20 g were challenged with *C. albicans* at a lethal dose (1 × 10^7^ cells). Experimental groups (1 × 10^7^ pfu JS and IS) and control groups (PBS, KM13 and anti-BSA) were administered 2 h after the infection intravenously, followed by a repeat treatment given at 24 h. The animal status was regularly monitored for a period of 14 days. The survival curves were analysed by Log-rank test using GraphPad Prism software version 5.00 (GraphPad Software, USA).

Mice were infected with 1 × 10^6^ cells of *C. albicans* by the intravenous route. 1 × 10^7^ pfu JS or IS was therefore administered intravenously 2 h after the infection, followed by a repeat treatment given at 24 h. PBS, KM13 and anti-BSA phage were served as negative controls. Mice were killed at 3 days post-infection for further studies.

Kidney samples from infected mice were immediately fixed by formalin and dehydrated with xylene, and then rehydrated through a graded series of ethanol solutions (80%, 90%, 100% ethanol) and embedded in paraffin. Tissue sections (5 μm) were mounted onto glass slides and stained with hematoxylin and eosin (H & E) or periodic acid-Schiff (PAS) using conventional staining methods. Slides were analyzed under light microscopy.

To assess the tissue outgrowth of *C. albicans*, the kidneys, spleens and hearts were collected, weighed and homogenized in sterile PBS with a tissue grinder 3 days after infection. *C. albicans* burdens were determined by plating dilutions of the homogenates on YPD agar plates, and incubating the plates overnight at 37 °C for colony formation, as described elsewhere^45^. The CFU were counted and expressed as the log CFU per gram of tissue.

### *In vivo* analysis of cytokine response

Before cytokine detection, the blood samples were collected on day 3 after infection and stored at −20 °C. The levels of IL-2, IL-10 and IL-17 in murine plasma were measured using enzyme-linked immunosorbent assay kits (Affymetrix ebioscience, USA) following the instructions of the manufacturer. Cytokine titers were calculated by standard curves.

### Statistical analysis

All *P* values of <0.05 were considered significant. Quantitative variables were analyzed by one-way analysis of variance (ANOVA). GraphPad Prism 5 software was used to perform statistics and graphs.

## Additional Information

**How to cite this article**: Dong, S. *et al.* Novel nanoscale bacteriophage-based single-domain antibodies for the therapy of systemic infection caused by *Candida albicans. Sci. Rep.* **6**, 32256; doi: 10.1038/srep32256 (2016).

## Supplementary Material

Supplementary Information

## Figures and Tables

**Table 1:** The effect of phage on blood characteristics in mice.

	WBC(10^9^/L)	LY(10^9^/L)	NE(10^9^/L)	MO(10^9^/L)	RBC(10^12^/L)	HGB(g/L)	PLT(10^9^/L)
PBS	10.7+7.6	5.6+4.7	4.3+2.3	0.7+0.6	7.1+0.3	150+95.5	94.3+10.2
KM13	14.1+3.1	3.5+1.5	7.1+1.0	0.6+0.2	6.5+0.3	194+10.4	104.7+100.1
*P* value	N/S	N/S	N/S	N/S	N/S	N/S	N/S

5 mice in each group and the results were expressed as the mean ± standard deviation. N/S, not significant.
