# Supplementary material for: Novel nanoscale bacteriophage-based single-domain antibodies for the therapy of systemic infection caused by Candida albicans
Source: Sci Rep. 2016 Aug 25;6:32256. doi: 10.1038/srep32256 (PMC4997605; doi:10.1038/srep32256)
Supplement: Supplementary Information [file srep32256-s1.pdf]

Novel nanoscale bacteriophage-based single-domain antibodies for the therapy of systemic infection caused by *Candida albicans*

Shuai Dong, [1] Hongxi Shi, [1] Donghui Cao, [3] Yicun Wang, [1] Xintong Zhang, [1] Yan Li, [1] Xiang Gao [1,2]* & Li Wang [1]*

[1]Institute of Cytology and Genetics, School of Life Sciences, Northeast Normal University, Changchun City, Jilin Province, P. R. China, 130024

[2]Key Laboratory of Molecular Epigenetics of MOE, Changchun City, Jilin Province, P. R. China, 130024

[3]Division of Clinical Epidemiology, First Hospital of Jilin University, Changchun City, Jilin Province, P. R. China, 130021

Correspondence and requests for materials should be addressed to X. G. (Email: gaoxiang426@163.com) or L. W. (Email: wanglee57@163.com)

Supplementary Figure S1

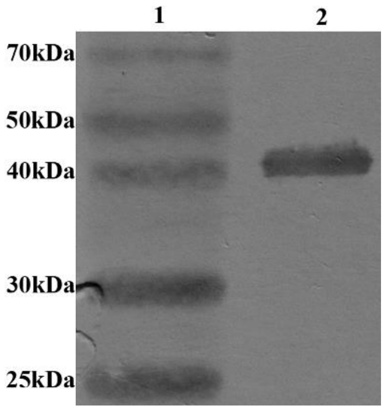

Supplementary Figure S1: Western blotting analysis of purified rSap2 recognized by anti-His tag monoclonal antibody. Lane 1: Protein Marker; lane 2: rSap2.

Supplementary Figure S2

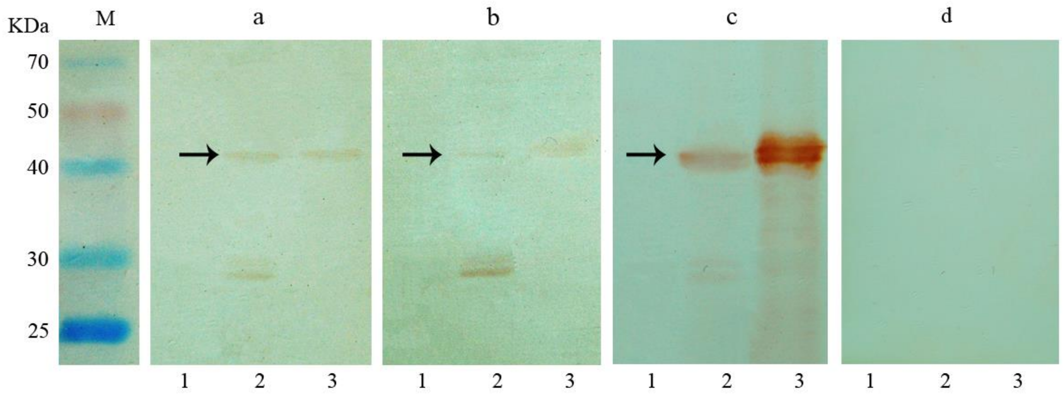

Supplementary Figure S2: Western blotting analysis of *C. albicans* preparations. Cell walls, total cell lysates and purified rSap2 were resuspended in PBS at the concentration of 10μg, and subjected to SDS-PAGE before blotted with JS (a), IS (b), anti-rSap2 pAb (c) and KM13 (d), respectively. M: Protein Marker. Lane 1: cell walls lysates. Lane 2: total cell lysates. Lane 3: purified rSap2. Arrows indicated the position of native Sap2 and rSap2.

Supplementary Figure S3

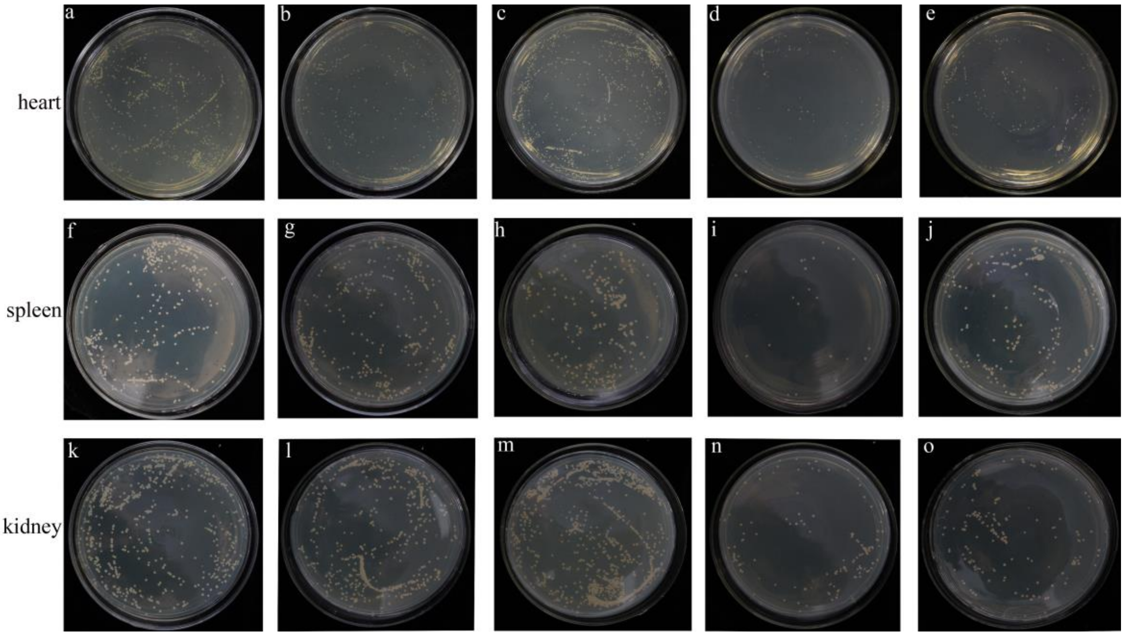

Supplementary Figure S3: Image of colony forming units plates. (a), (f), (k) PBS groups. (b), (g), (l) KM13 groups. (c), (h), (m) anti-BSA groups. (d), (i), (n) JS groups. (e), (j), (o) IS groups.

Supplementary Table S1

CLONE	Amino Acids	Polar Amino Acids (N,C,Q,S,T,Y)	Hydrophobic Amino Acids (A,I,L,F,W,V)	Isoelectric Point
JS	52	23 (44.2%)	13(25%)	10.228
IS	52	32 (61.5%)	11(21.2%)	9.047

Supplementary Table 1: The characteristics of amino acids in CDR regions.
